# A qualitative exploration of the mental health challenges and therapeutic needs of Canadian correctional workers

**DOI:** 10.3389/fpsyt.2022.1004143

**Published:** 2022-10-25

**Authors:** Elnaz Moghimi, Yuliya Knyahnytska, Yiran Zhu, Anchan Kumar, Alexander Knyahnytski, Charmy Patel, Mohsen Omrani, Cory Gerritsen, Michael Martin, Alexander Ian Frederic Simpson, Nazanin Alavi

**Affiliations:** ^1^Department of Psychiatry, Faculty of Health Sciences, Queen's University, Kingston, ON, Canada; ^2^Centre for Addiction and Mental Health, Toronto, ON, Canada; ^3^Faculty of Health Sciences, Queen's University, Kingston, ON, Canada; ^4^Faculty of Arts & Science, University of Toronto, Toronto, ON, Canada; ^5^OPTT Inc., Toronto, ON, Canada; ^6^Correctional Service of Canada, Ottawa, ON, Canada; ^7^School of Epidemiology and Public Health, University of Ottawa, Ottawa, ON, Canada; ^8^Faculty of Health Sciences, Centre for Neuroscience Studies, Queen's University, Kingston, ON, Canada

**Keywords:** correctional workers, internet, mental health, online, psychotherapy, public safety personnel, mental health disorders

## Abstract

**Purpose:**

Correctional work is described as a high-stress environment associated with increased prevalence of mental health disorders in employees. Identifying appropriate healthcare services necessitates investigating the mental health challenges and needs of correctional workers (CWs).

**Methods:**

Individual interviews (*n* = 9; 5 M and 4 W) and a mixed gender focus group (*n* = 6; 3 M and 3 W) were conducted to gather a general sense of the mental health landscape. Data were analyzed to develop a targeted and comprehensive question guide for gender-specific focus groups (*n* = 14 unique participants; 6 M and 8 W).

**Results:**

Eight themes emerged from the gender-specific focus groups. Themes focusing on work culture described the negative repercussions of job stress and the inability to discuss challenges openly due to confidentiality concerns and feelings of seclusion associated with the CW profession. Men were more likely to be subjected to physical violence and women to emotional and sexual harassment from staff and inmates. Themes related to mental health care described the benefits and limitations of the current services and digital mental healthcare. Stigma and accessibility were notable treatment barriers. Lastly, sector-specific therapy was seen as an important component in enhancing engagement and therapist interaction.

**Conclusion:**

The study demonstrates the interconnection between work culture and CW mental health that needs to be acknowledged when addressing mental health care.

## Introduction

Correctional workers (CWs) are public safety personnel (PSP) responsible for the security, safety, and provision of services for staff and inmates within prisons, jails, courthouses, and correctional centers (1). CWs often report highly stressful work conditions and frequent exposure to potentially psychological traumatic events (2). The demanding nature of this profession may partly explain why the risk of suicide and mental health disturbances is typically greater in CWs than in the general population (1, 2). In a survey of correctional service employees in Ontario, Canada (*n* = 1,487), 59% of correctional officers met the diagnostic criteria for one or more mental health disorders (3). Furthermore, women were significantly more likely to meet diagnostic criteria for psychiatric disorders than men (3). The repercussions of untreated occupational stress and mental health concerns can also impact physical health and interpersonal relationships and increase familial anger, stress, and strain (4).

Although different stressors can contribute to the overall risk of mental disorders in CWs, the genesis of specific mental disturbances seems to stem from various aspects of the work environment. Commonly reported contributors include safety concerns, physical assaults and verbal threats, bullying and harassment, witnessing murder or suicide attempts, and dealing with constrained work conditions such as high workload, understaffing, and overcrowding (1–6). The amalgamation of these stressors can contribute to CW distress and mental un-wellness, which can lead to suicidal thoughts and behaviors (7). In a systematic review of mental disorders amongst CWs, post-traumatic stress disorder (PTSD) was strongly associated with physical violence and work injury (1). Major depressive disorder (MDD) and anxiety disorder were strongly associated with low perceived administrative support, job satisfaction, and perceived social value of the work role (1). In a recent qualitative study, the cultural norms of discouraging behaviors or reactions that may be perceived as weak or emotional may also contribute to the high prevalence of mental health disorders in CWs (8). Many CWs also find that there is limited mental health support (9). Moreover, systematic and individual factors such as stigma, repercussions at work, and understaffing are reported barriers to treatment-seeking (10). How these factors are associated with specific mental health disorders and treatment-seeking requires further exploration.

The lived experiences of CWs can provide rich and detailed insights into the dynamics of the mental health and treatment-seeking challenges they face in their work environment. The current study is the first phase of a larger randomized controlled trial (RCT) that will investigate the efficacy of online psychotherapy in this population (11). The virtual platform (Online Psychotherapy Tool; OPTT) offers electronic cognitive behavioral therapy (e-CBT), the first-line psychotherapeutic treatment for many mental health disorders (12–14). The current study explored CWs' personal experiences with their mental health concerns and care delivery. In-depth 1:1 interviews and focus groups focused on how CWs interacted with prisoners and colleagues, their work conditions and conflicts, mental health challenges they faced, perceptions of mental health resources and support, and their attitudes toward mental health care. Participants were also asked to provide feedback on previous modules for PTSD and depression available on OPTT (11, 15). The findings of the current study will guide the development and evaluation of e-CBT programs for CWs in the subsequent phases.

## Methods

### Participants and study design

The study recruited current or previous correctional employees in Canadian federal and provincial facilities in Ontario, Canada through social media, flyer advertisements, and clinician or self-referrals. Telephone screening was conducted by a trained research assistant (RA) to confirm eligibility. The RA explained the study to participants (*n* = 21; 10 men and 11 women), answered any questions, and obtained informed consent to participate in interviews and focus groups. The study used individual interviews and five successive focus groups. The interviews were ~60 min in length and the focus groups were 90–120 min. All open-ended question guides were pilot tested before use. The focus group sample sizes were between 3 and 6 participants to create an intimate setting for experiences to be shared in depth (16). All interviews were conducted via a secure online video platform and participants were given the option to turn their webcams on or off to preserve their anonymity. Prior to commencing the interviews and focus groups, the participants were informed that they could leave at any time, that they did not need to answer all the questions, and that this was a confidential conversation. Participants received a $75 Amazon gift card for their time. The study was reviewed for ethical compliance by Queen's University Health Sciences and Affiliated Teaching Hospitals Research Ethics Board; File Number 6029966.

### Individual interviews

Semi-structured 1:1 interviews of a mixed-gender sample of CWs (*n* = 9; five men and four women) were conducted by trained RAs (*n* = 3). The 36-item question guide explored the participant's work in the correctional facility (four questions), violence, harassment, and other challenges experienced at work (twelve questions), support networks (six questions), past psychiatric history (four questions), the relationship between mental health and correctional work (six questions), and thoughts about online care (four questions). Participant responses were recorded by RAs through type-written notes.

### Mixed-gender focus group

A purposive sample of individuals who completed the interviews (*n* = 6; three men and three women) participated in the focus group. The semi-structured question guide explored the level of interaction with prisoners, types and frequency of violence at work, work conditions, socioeconomic factors, mental health challenges, care resources and frequency of use, preferred methods of support, and attitudes toward online care. Participants were also presented with previously validated modules for PTSD and depression for the general population through short video screen captures (17–19) and a brief explanation of how the therapy sessions would be structured. Participant responses were type-written by RAs conducting the focus group (*n* = 3). A member check of the individual interviews and focus group was conducted (20). Here, participants and interviewers were emailed a summary of the emergent themes and asked to provide written feedback.

### Gender-specific focus groups

Data from the individual interviews and mixed-gender focus group informed the development of four semi-structured and gender-specific focus groups (MFG1, MFG2, WFG1, WFG2). Five individuals (MFG1 *n* = 3; WFG2 *n* = 2) who had agreed to participate did not attend the focus groups due to reasons unknown (*n* = 4) and not feeling comfortable sharing their experiences (*n* = 1). The final sample size for the men's focus groups were, MFG1, *n* = 3; MFG2, *n* = 4 and for women were, WFG1, *n* = 6, WFG2, *n* = 2. One male participant had to leave within the first half-hour of MFG1 and agreed to join MFG2. Data from his participation in both focus groups were analyzed. The question guide comprised five open-ended questions and additional probes exploring participants' thoughts and opinions on the previously generated themes and how therapy could address them, strategies to make therapy more easily available to CWs, and general opinions of a CW-specific module that would be available on the OPTT platform. Participants were also asked to discuss how their gender impacted their work experience and how therapy could be designed to address gender-specific needs. The focus groups were conducted by a postdoctoral fellow and three RAs.

The screen-recorded sessions were transcribed *verbatim* by the postdoctoral fellow conducting the focus groups and the resulting transcripts were proofread by an RA involved in the focus groups. To protect anonymity, identifying information was omitted, and random numbers along with gender symbols (W = woman; M = man; e.g., PW1, PM1) follow the presented quotes. Where needed, the quotes were edited for correct grammar and spelling while maintaining their meaning and tone. A member check was also conducted and all study participants (*n* = 21) were emailed a summary of the final themes and asked to provide their written feedback.

### Data analysis

The consolidated criteria for reporting qualitative research (COREQ) checklist was used to report qualitative findings (21). Textual data from the individual interviews and mixed-gender focus groups were analyzed using systematic text condensation, informed by Giorgi's psychological phenomenological analysis methods (22). This descriptive and exploratory method is frequently used for thematic cross-case analysis of different types of qualitative data to uncover meaning behind a person's experience (22). The themes centered around the lived experiences of CWs with their mental health and how it may have been impacted by their profession. The data were analyzed by a co-author (YK), a clinician-scientist with experience in qualitative analysis methods. The emergent themes were presented to the research team for discussion and finalization.

Data from the gender-specific focus groups were analyzed using inductive thematic analysis (23) by the postdoctoral fellow and RA who conducted the interviews. Thematic analysis was conducted (24). Initially, the transcribed screen recordings from each focus group were uploaded onto NVivo 12 (25), and open codes were generated that captured prevalent ideas amongst CWs. These codes were then organized into primary, secondary, and tertiary categories, and a codebook of themes was generated. With the analysis of each subsequent focus group, the codebook was modified and revised to capture new insights generated. The themes were then discussed amongst the research team and revised where necessary.

## Results

### Participant demographics

The average age of demographic questionnaire respondents (*n* = 19) was 43.47 years (SD = 11.18). The majority were white (17/19, 89.4%) and married. In addition, most possessed a bachelor's degree (12/19, 63.2%) and were correctional officers (12/18, 66.7%). The average years of employment were 13.86 years (*n* = 18, SD = 9.64). Most participants continued to work full time (13/19, 68.4%) and had an annual income between $75K-99,999 (12/19, 63.2%). Lastly, most participants reported that they were diagnosed with a mental health condition (17/19, 89.5%). Additional demographic details are outlined in Table 1.

**Table 1:** Summary of participant demographics.

	**All participants**
**Gender (*n*, %_**totalres**_)**	
Man	10, 47.6
Woman	11, 52.4
**Age (*n*, Mean, SD)**	19, 43.47, 11.18
**Ethnicity (*n*, %_**totalres**_)**	
White	17, 89.4
Other	2, 10.5
**Marital status (*n*, %_**totalres**_)**	
Married	10, 52.6
Divorced	4, 21.1
Single	3, 15.8
Other	2, 10.5
**Number of children (*n*, Mean, SD)**	19, 1.47, 1.5
**Highest education (*n*, %_**totalres**_)**	
Bachelor's degree	12, 63.2
College diploma and high school diploma	6, 36.8
**Employment years (*n*, Mean, SD)**	18, 13.86, 9.64
**Employment status**	
Full time	13, 68.4
Time off work	4, 21.1
No longer employed	2, 10.5
**Annual income (*n*, %_**totalres**_)**	
$50K-74,999	3, 15.8
$75K-99,999	12, 63.2
$100K+	4, 21.1
**Self-reported mental diagnosis (*n*, %_**totalres**_)**	
Depression	4, 21.1
Anxiety	3, 15.8
PTSD	5, 26. 3
Multiple diagnoses	5, 26.3
No diagnosis	2, 10.5

n = sample size; %_totalres_= percent of total respondents; SD = standard deviation.

### Initial interviews and mixed-gender focus group

#### Themes

Six themes emerged which described the correctional workers' roles and responsibilities; relationships and interactions; coping; preparedness; work environment; and mental health needs. The themes have been summarized in Table 2.

**Table 2:** Summary of initial interviews and mixed-gender focus group themes.

**Theme**	**Subthemes**
Roles and responsibilities	(1) Hidden and unrecognized emotional labor (mental) from overlapping roles and additional tasks
	(2) Negotiating roles (guard vs. therapist vs. nanny vs. clerk vs. housecleaner)
Relationships/Interactions	“Single warrior” vs “brotherhood”
	Distrust and frustration with “Big Brother watching”
Coping	Low support and care from administration and management and “feeling like you don't matter”
	Detachment as coping (to protect themselves emotionally from inmate and staff-related trauma)
	Work around and “try to ignore dumb rules from management”
Preparedness	“Reality is different from training…[training] is inadequate to deal with reality”
	“Threw binders” type of training (using textbooks and making little practical sense)
Work environment	“Variable depending on the amount of chaos”
Mental health needs	Accessibility (time, money, knowledge of resources)
	Attainability (can actually dedicate time)
	Regularity and support (managerial, colleagues)

#### Member check of individual interviews and mixed-gender focus group themes

The themes were sent to 12 individuals (nine participants and three interviewers). Three individuals responded (two interviewers and one participant). Non-respondents (*n* = 9; one therapist and eight participants) were assumed to accept that the data were valid and reflected reality. The sole participant, a man, expressed, “this will sound very short and lazy, but that hit the nail on the head and I can't add much more.” Both interviewers found that the themes and descriptions resonated with their experience in the interviews and reflected the mental health needs and concerns that were discussed.

### Gender-specific focus group

#### Themes

Eight themes and 21 subthemes emerged from the gender-specific focus group responses. The themes were divided into two categories that described the correctional work culture and mental health care (Table 3).

**Table 3:** Summary of gender-specific focus group themes.

**Work culture**	
**Theme**	**Subtheme**
Job stress	Trauma and violence
	Multiple roles at work
	Relationship with management
	Long-term effects
Seclusion of profession	Hiding weakness
	Disconnect between work and personal life
	Job transparency
Gender differences	Violence
	Harassment of women
**Mental health care**	
**Theme**	**Subtheme**
Workplace mental health support	Institutional programs
	Peer support
	Workplace training
Treatment benefits	Normalization
	Coping mechanisms
Treatment barriers	Accessibility
	Stigma
Therapy needs	Proactivity
	Therapy design
Online modules	Convenience
	Engagement
	Therapist interaction

### Category: Work culture

#### Theme: Job stress

##### Subtheme: Trauma and violence

Participants described frequent exposure to trauma and violence: “I don't think I know one officer that hasn't been through something traumatic or violent or whatever it is. A lot of their deep-seated mental health issues stem from issues of that nature” *(PW16)*. CWs were aware that enclosing individuals in a confined space would result in a natural restlessness. However, what distinguished correctional work from other PSP sectors was:

Where we work, there's no place to go. If there's an incident, you stay where you are, and the perpetrator goes out to the hospital and comes back to the same unit and you have to deal with the same guy that you had issues with. Paramedics will get to an incident and they won't see them again, ever, the people that they fix. People that we try to fix, we see them again and again and again. Some of them for ten, fifteen, 20 years *(PM4)*

##### Subtheme: Multiple roles at work

The work environment expected CWs to assume different roles, even if they were not trained in that area: “sometimes I get a feeling that our role has merged into semi-counselors but without all the education,” *(PW12)*. Another participant stated that there's “not enough support given to perform tasks outside of your job description. That is definitely something that has frustrated me” *(PM1)*. Many CWs described the general work as monotonous and that wearing multiple hats usually occurred during incidents:

You have your routine, your feed, you do your meds, walks, regular things over and over and over again and that gets really monotonous and tedious. But then when something does happen, you become all of the above. You are the fire department, you are the paramedic, you're the social worker, you're the police officer. You have all these different hats that you may be required to wear at any given time throughout your day or your week. So I think that can become a little strenuous, having to be everything all at once. *(PM3)*

##### Subtheme: Relationship with management

Participants described having different experiences with different managers: “I've had some managers that are really supportive and really great and I've had others that are absolutely horrible” *(PW13)*. Another participant described differences in the level of management: “some people that work in senior management have never worked in a day in the life of a correctional officer” *(PW14). One* of the criticisms was that during incidents, staff mental well-being was not prioritized:

If there's an incident that happens, the first thing you hear is to make sure to cross your T's and dot your I's instead of, how are you guys doing? Did you guys survive that swing or [the offender] trying to kill you? Or did you make sure that your documentation is right? *(PM4)*

Mental health views were reported to differ amongst managers and staff who had been in service for a longer period of time: “If you've got a manager that might be coming around 30 years, in my firsthand experience, I hear that mental health isn't really pushed. It's kind of like an old guard mentality with that person,” *(PF16)*. Mental health services promoted by management also seemed disingenuous: “it's kind of like they do it because they have to do it, not because they want to do it” *(PM3)*. These feelings may be due to a lack of perceived care and concern: “They put up all the posters but I've never had a manager stop me and ask me actually how I'm doing” *(PM2)*. CWs also indicated a disconnect between management and the frontline: “there was no meaningful dialogue which again adds to that frustration” *(PM5)*.

##### Subtheme: Long-term effects

Many CWs experienced the slow and cumulative effects of workplace stress and violence in the long term: “mine was insidious...it just accumulated to the point where my family doctor said, you can't do it anymore…I don't know how really to describe it other than it was a slow development of losing myself” *(PM5)*. Correctional work was described as changing behaviors over time: “it completely changed me. Where I wasn't this aggressive person, I became completely aggressive” *(PM6)*. The negative effects also extended to other areas of a person's life: “I love what I do…However, it's taken its toll personally, professionally, with relationships, with mental health. It's taken its toll in so many ways,” *(PW16)*. Emotional detachment was seen as a way to cope with traumatic experiences and burnout: “it's a quiet survival mechanism” *(PM5)*. Another participant found that positive behaviors outside of the work environment were not beneficial and resulted in emotional detachment at work:

I'm a very caring, courteous, polite person and I tried to maintain that inside of that environment. That creates a lot of emotional labor because it's very difficult for me and was sort of the cause of my mental injury, that conflicting emotion in there. Obviously, I did cope with it by emotionally detaching. *(PM1)*

#### Theme: Seclusion of profession

##### Subtheme: Hiding weakness

CWs found that expressing concerns at work was associated with weakness: “you're supposed to be strong and in control and to say that having an incident affect you is showing weakness” *(PM3)*. The correctional culture was described as “the culture [of] bottle it up” *(PW16)* and the “culture of just suck it up and carry it on” *(PW15). One* participant reported that “a bravado exists amongst both sexes or genders” *(PM2)*. This culture was also described as one frequently practiced by the older generation of CWs with an influence on younger employees:

They [new officers] see or they hear about these older officers and how they act in this sort of macho environment way, this toxic masculinity way, and they try to act in that way too to fit in. And I think that's a big issue. *(PW13)*

A fear of judgement was listed as a reason why CWs refrained from addressing mental health needs:

I find with trying to access all of that stuff is the fear that someone that you know, [who] is one of those stereotypical macho guards will literally call you out for it and make you feel like garbage because you need help when you know that they're almost as broken, if not more broken than you are. Just the façade is very intimidating. *(PW12)*

##### Subtheme: Disconnect between work and personal life

Most CWs found a disconnect between their identities inside and outside work:

When you leave the gate, leave the institution, you turn the [correctional worker] off a little bit and then you turn into the husband, or the father, or the friend, or whatever role you're going to have that you name on your rest days. It's a little bit of a disconnect. *(PM3)*

Some participants also expressed that it was hard to disconnect from work and that it had an impact on their personal life:

I was always on the edge. We're always in a state of readiness, right?...This is why when you see a lot of the guys going to the restaurants, they always have their back to the wall. Because you always have that state of readiness. *(PM6)*

Many CWs also reported that control was necessary at work but became problematic in their personal lives:

Every aspect of somebody's [offender] life is controlled by us in some regard…it's hard for us to lose control…I know when I go home and my wife and I argue or bicker about something, it's very hard for me sometimes to relinquish that control because I'm so used to having it. *(PM2)*

Many of the participants found that those outside corrections did not understand the nature of their job: “they [friends and family] don't get that your job is literally so stressful that your anxiety levels are at 100 most of the time,” *(PW12)*. Some CWs described the public as being in the dark regarding the details of the CW profession, making it hard for them to share their work experiences:

People who are not in corrections don't understand what life is like inside and so it's hard to be able to share any of that experience or for them just to have a basic understanding of what everyday life is like there. *(PW11)*

The inability to confide in family members was reported to be guilt-inducing: “I carry a lot of guilt, you know opening up to my family about things like that because I don't want to traumatize them and vicarious trauma is a real thing,” *(PW16)*. Feelings of isolation and exclusion were also apparent in the participants:

[We're] behind the wall, nobody really knows about us, nobody really has talked to us, we haven't really been included in a lot of things. If they talk about a law enforcement day, correctional officers are rarely included. We're typically excluded from most things. *(PM2)*

##### Subtheme: Job transparency

Many CWs expressed a lack of clarity from the organization regarding their work-related duties: “they kind of try to leave out the dangers and the long-term effect of what you will be seeing all the time,” *(PW12)*. The lack of transparency also added to CWs feeling unsupported in the workplace: “I think the negativity, the lack of support, lack of opportunities. I think that has been the most detrimental to my mental health, than it has been dealing with inmates stabbing each other” *(PW16)*. Some suggested that the institution should disclose the dangers associated with the profession and offer peer support:

I think right at the beginning when somebody is hired that I think we should be really honest with the person that's being hired that these are the things you're going to go through. Let's not make it a pink sky, it's gonna be rough. You're gonna go through these things and maybe have people like us talk to them and have discussions with new recruits to explain these are the things you're gonna go through at different times in your career. *(PM6)*

#### Theme: Gender differences

##### Subtheme: Violence

Aggression was reported to vary by gender: “men experience a lot more physical violence and women experience sort of a more psychological or emotional violence” *(PM2)*. With respect to employee duties, the only gender difference reported by males was related to strip searches: “besides having to do all of the strip searches because I'm a male, there's not really much in the way that differentiates the strain between or the difference between a male and a female correctional worker in my mind” *(PM1)*. Strip searches made men CWs more vulnerable to physical assaults by inmates: “uses of force stem from strip searches and incidents like that…I can't really think of a time when female officers were attacked. I can think of multiple times per month where male officers were attacked” *(PM3)*. Gender differences reported by men CWs also focused on the motherly nature of women CWs that could de-escalate potentially violent situations:

Women are able to speak with inmates on a more mother-to-child level than perhaps males do. I find a lot of women are really great at talking down or diffusing the situations where with some men, I think ego tends to get involved and it becomes a pissing match. *(PM2)*

##### Subtheme: Harassment of women

Women CWs were vocal about workplace gender discrimination - in the form of emotional and sexual harassment - and found it to be integrated into the culture:

If you're a female working in a male jail, you're basically getting it from all sides. You're getting disgusting comments from your coworkers and from the inmates. It's a different culture because you're a female and you're weak. It's that whole thing of females in roles that are traditionally male. It's another one of those crappy parts of the job. *(PW16)*

One participant also pointed out that men are unaware of how women experience the workplace:

I've had the male staff not even recognize how different their experience is… they'll walk past and hear an interaction and they'll come up after saying, “oh,” completely unaware of the challenge of that sort of emotional labor that we have to deal with. *(PW12)*

The constant need for the women to set boundaries with colleagues and offenders was described as emotionally taxing: “I find that the whole limit setting and boundaries thing with both offenders and fellow [CWs] to be one of the most draining things that I have to go through consistently every day” *(PW10)*. When asked about the perception of training programs to address workplace harassment, one participant stated: “It's mandatory training, like do this stupid little quiz and then give the manager my certificate” *(PW10)* and another woman CW added, “I think the top really doesn't want this to be an issue” (*PW4)*. Many of the women described hypersexuality in their colleagues to be a result of the work culture:

“I actually wonder if some of the hypersexuality of some of the female officers with male officers is because that's their defense…Some of it might be stressful, but I think some of it is that toxic male culture where, you know, every woman is a notch in the belt and then some of the women are like, ‘well, if it's good for the man, then it's good for me, and I'm gonna do it.”' *(PW4)*

Another participant added, “yeah, and for a lot of women, I wonder if it's a way of trying to fit [in] or gain approval of some of the male officers,” *(PW10)*. When the women were asked if they prefer to work in an all-women institution, most said no:

Women are just a lot more manipulative and try to get under your skin a lot more. To me, they're a lot more crazy, whereas with the men, for the most part, what you see is what you get. Yeah, you have guys that are trying to be manipulative but I find they're not as good at it as the women would be. I find that women coworkers alone can be really difficult and I couldn't imagine being in a whole prison of women. *(PW13)*

### Category: Mental health care

#### Theme: Workplace mental health support

##### Subtheme: Institutional programs

The limitations of mental health services offered by institutions were described by participants: “mental wellness is talked about constantly in everything we do and then these services are so limited that it just feels like lip service” *(PW15)*. Although some of the mental health programs offered by institutions were seen as helpful, participants expressed many drawbacks associated with the therapy-related services, namely, “it doesn't even scratch the surface of how traumatic the things you can see and how much that's going to affect you, even in your every day” *(PW12)*. The number of sessions offered was viewed as insufficient in addressing CW concerns: “the [program] offerings are so limited that by the time you've made a connection with somebody who understands your work and understands what's going on with you, you're out of sessions” *(PW15)*. Many participants pointed out that the help was not specific to their profession: “I find that through our [program] there is a lot of access, but the person you're getting isn't necessarily catering toward what we do” *(PM3)*. Sector-specific programs were also seen as necessary: “not having anybody on the [program] that is an officer…if they don't come from an officer background, it makes it hard to make that connection with someone who might work in admin or finance, you know?” *(PW14)*.

##### Subtheme: Peer Support

Peer support was widely seen as a positive addition to mental health support: “peers were a big support group for us” *(PM5)*. Moreover, positive relationships in the workplace were essential in managing many stressors and mental health concerns: “if I didn't work with [colleague] I probably wouldn't last this long…I think if we were working with somebody that you cannot trust and we've seen it before, we've seen people freeze in situations” *(PM4)*. Peer support was also viewed as improving treatment-seeking:

Sometimes you need that guy to say, ‘you know what, I know you're going through some tough times, come with me,' you know? And have that friend or that guy that works with you, just to help you go through it. *(PM6)*

Another CW indicated that programs with peer support contributed positively to her relationship with colleagues:

I found it kind of created an environment where people could open up a little bit more and have that sense of trust. I could see how it would really help when it's another [CW] because they know what you're going through. *(PW14)*

A structured peer support system was described as a way to reduce feelings of isolation and to enhance debriefing, “an opportunity to debrief as a team makes you feel less alone in this situation, it's validating, it's got growth, there's personal development that comes from that” *(PW15)*.

##### Subtheme: Workplace training

The need for workplace mental health training was expressed by most participants. Although the current training was somewhat helpful, there were areas of improvement disclosed. Some participants expressed that the training programs do not reflect their work experience, which reduces the authenticity of the services offered:

I don't think it's authentic. I think it's like someone sitting behind a computer making this PowerPoint and has no idea what I'm doing on the frontline…I think where the information comes from is so out of touch with us on the front lines that it doesn't really do anything. *(PF16)*

This inauthenticity was also reflected when CWs explained their perceived intention of the institution and management behind the trainings:

Every year, we have to take this 5-min online course about workplace violence and harassment. But nobody really pays attention to that, nobody puts effort into it, nobody reads the material.…[The institution believes]: Okay, we have some harassment, so we'll do a harassment training, people will take it, everything will be solved. They [institution] don't really want to admit the problems we have and actually do the work to fix them. *(PF16)*

#### Theme: Therapy benefits

##### Subtheme: Normalization

A notable benefit of therapy was its ability to normalize the experiences of CWs: “the most helpful thing was just having someone kind of normalize my thoughts and feelings and things that happened to me” *(PF13)*. As feelings of isolation were frequently reported by CWs, therapy provided comfort that they weren't alone in their mental health struggles:

It gave me the ability to go back to work and realize that this is something that everybody struggles with and we just need, as a collective group, to do better at talking to each other about it and therapy really helped me do that. *(PM1)*

A space to talk openly was seen as an important component of normalizing therapy: “having someone to just kind of bounce your experiences off of and also just kind of to let you know that it is normal, that is really helpful” *(PM3)*.

##### Subtheme: Coping mechanisms

CWs who had previously received therapy described its effectiveness in teaching positive coping mechanisms. Participants also shared how therapy improved relationships with family members: “therapy was helpful to learn how to recognize when I was feeling overwhelmed and rather than take it out on my family, to remove myself from the situation so that things didn't get out of hand,” *(PF11)*. Therapy was also described as providing skills to help participants relax:

The PMR [progressive muscular relaxation], the imagery, and the mindfulness. When I feel like I can't cope or if I feel like things are really starting to get out of control, I use those to help me and my mind just run through it naturally. *(PM1)*

The same participant also expressed the benefits of these strategies in his personal and professional life:

You're a different person when you're at work. You tune into what's going on and you're very focused and hypervigilant, which is good for your job but bad for us as people. I'd like to be able to have something that can help my mind relax a little bit more or my body relax a little bit more inside the prison system so that I don't feel so tense and uptight all the time. *(PM1)*

#### Theme: Treatment barriers

##### Subtheme: Accessibility

Easier access to care was viewed as a good preventative measure: “just the need for easy access and mental health care and a therapist. That would be nice, beforehand and before getting to the point where I was,” *(PM1)*. Due to the nature of correctional work, it was also important for programs to provide faster access to those seeking care:

When I call [program], and they're like, ‘well, it'll be two days and someone will get a hold of you,' and two days goes by, I'm immediately like, ‘am I forgotten about? Do they not care? Who am I getting put with?' And then I just go through like worst case scenario because my job is all what-ifs. *(PM2)*

##### Subtheme: Stigma

Workplace mental health stigma was frequently listed as a barrier for CWs to seek help and talk about their mental health challenges:

I think there's still a huge stigma attached to mental health. Even myself, it took me years to step up. And I knew I was having issues but I was just like, ‘I can get through it, I can get through.' And finally, I wasn't. I broke down. *(PM6)*

Because some interventions were offered in a public environment, it prevented some CWs from accepting them: “the sad thing is because they offer it in a public environment, everybody's gonna decline or most people are gonna decline even if they might not have declined in another situation,” *(PF4)*. Stigma contributed to a fear of judgement within the workplace culture, which discouraged CWs from receiving care. When the idea of an onsite mental health care office emerged, one participant expressed:

The stigma is still there and as much as we want to say that it's not or it's changing, I think that some people will still have that fear, like ‘ok, I'm going to go there [onsite office for mental health care], what are my people going to think?' *(PF14)*

One participant described the fear of work repercussions which prevented CWs from seeking help:

A lot of people worry that you're gonna be labeled with something, that you're gonna go and talk to somebody and then a doctor is going to recommend that you take some time off…and while you're off, people at work are gonna be seeing you taking time off, not dealing with what they're dealing with…and the stigma of waiting and getting yourself right while they [coworkers] have to deal with it [mental health challenges] is what's really difficult for a lot of people to seek help. *(PM2)*

Normalizing mental health and addressing the “macho culture” *(PF10)* was seen as an important way of reducing stigma, “I don't know how we can do it, but I think we need to normalize mental health as much as we do physical health…we need to help staff realize that mental health problems are still health problems,” *(PF4)*. Some CWs were hopeful that the younger generation will contribute to greater awareness and changes in workplace mental health stigma:

I know officers who have said, ‘I'm broken and I'm getting help for this and I'm doing whatever treatment or therapy,' and they speak openly about [it] and that gives me hope for a new generation and a new future for corrections. *(PM4)*

#### Theme: Therapy needs

##### Subtheme: Proactivity

Participants described a reactive work culture and many expressed the need for proactive mental health interventions:

The service, in general, is very reactive. It's not just mental health, it's not just taking care of their employees, it's in all aspects. I think that a really big part of what they need to break is trying to figure out how to fix things or stop problems before they happen. *(PF16)*

Another participant described, “you know, everything was after the fact. There's nothing that was proactive” *(PM5)*.

#### Subtheme: Therapy design

Many aspects of therapy design were discussed amongst the participants. The benefits and limitations of individual and group therapies were described by participants. One of the benefits of individual therapy was that there was no judgement: “You just feel like you can say what you need to say without judgement” *(PF14)*. Another participant who preferred individual therapy said: “it's really hard as a [CW] to open up in front of a group of people…It's one thing to do it [with] other staff…It is way harder to do it in front of people that you know,” *(PM2)*. At the same time, confidential group therapies were also viewed positively: “[CWs] can get together and have those discussions in a form that's safe and shared by everyone” *(PF11)*. Another participant indicated that sharing experiences can be “kind of an education” *(PM6)*. The majority of participants believed that individual therapy could be the first line of treatment, followed by group therapy:

I think I'd like individual [therapy] maybe initially. [Then] I think developing a support group of peers who literally are going through the exact same thing, or very similar anyway. A one-on-one with someone who has maybe a more psychotherapy background can provide actionable tools. *(PF10)*

Participants had variable opinions on online and in-person therapy. Most preferred in-person delivery and felt they would be more committed to it because “not everyone has the time or the structure in their life to necessarily do things online…not everybody's self-driven” *(PF4)*. Some attributed their preference for in-person delivery to the social nature of their work: “my interactions with people are always face-to-face with eye contact and I find that is an important part of relaying your proper mental state - is to be able to look at somebody and say that” *(PM1)*. Some participants also described the impersonal aspect of the online delivery method: “there's kind of a little bit of a disconnect when you do something over zoom, or teams, or via phone. There's that lack of personal aspect to it,” *(PM3)*. In contrast to most CWs, one participant described the benefit of writing things down in online therapy: “I know I feel like I have a hard time articulating from my head to verbally. So sometimes I write it down and reassess it, just to get a clear picture of what I'm trying to say,” *(PF14)*. Having therapy that acknowledged CWs' experiences was also a way to make them feel cared for: “I think having firsthand knowledge and experiences integrated into the therapy makes it feel like they give a shit about what we have to say and about how we want to make it better for the next person” *(PF16)*.

Lastly, the majority of CWs expressed the importance of working with therapists who had a strong background in corrections: “we should have therapists who know what our work environment is and the baggage that you can take from there into your everyday life” *(PF4). One* of the reasons experienced therapists were necessary was because: “they [therapists without correctional experience] really can't empathize with us very well. They understand trauma, I believe, or they understand trauma from their point of view, but it's not perhaps the same trauma that we go through” *(PM2)*. Working with experienced therapists was also viewed as a way to enhance treatment-seeking: “if I knew right away that I was going to talk to so had a background in it [corrections] rather than just somebody from the street, I would be personally more inclined to call” *(PM2)*.

#### Theme: Online CW-specific psychotherapy modules

##### Subtheme: Convenience

The online modules were viewed as a convenient form of therapy by some:

I like the idea that you can take it in chunks if you need to, you know. Some weeks maybe I'd sit there and do the whole thing and the other ones maybe I'd do it over a couple of days. *(PM5)*

The time flexibility of the platform was also appealing to a participant who was unable to concentrate for long periods of time due to his mental health injury. However, concentrating on a screen was challenging for another participant: “it's just the fact that I have to sit there and concentrate on a screen instead of just talking with someone. And I have difficulty concentrating, so that's my issue” *(PM6)*. The accessibility of the therapy was also a positive feature for some CWs:

It sounds like it would work. It would allow people to access mental health care from their homes, which would be a little bit more comfortable for them if they're having a hard time with the social aspect of life. *(PM1)*

Another CW also acknowledged the program's broad reach: “let's say, 15% are going to do it, that's wonderful that you've reached that 15% that you wouldn't before” *(PM3). One* participant summarized the advantages and disadvantages as follows:

So advantages, definitely would be, it's online, if you have an internet connection, you can access it. You can go back and review the material. You can kind of do it at your own pace, kind of like an online course sort of thing…But I think the disadvantage is if someone is pretty far into a spiral of their mental health, they may not want to take the time out of their day [or] give a crap in general. They may not understand what the material means, or they might be an older person that's not computer savvy or things like that, that might need more traditional talk therapy or go in-person or something like that *(PF16)*

For some, the exercise due dates had drawbacks:

Some people do very well with a due date and other people would really struggle with it and I know I'd really struggle if it was like, ‘here's your PowerPoint, you have to check through and do the homework and click on it and send it back,' and one week you'd get great work from me and the next week you'd get pretty rough work from me. *(PM2)*

##### Subtheme: Engagement

Due to the novelty of the online programs, many of the participants were willing and open to trying it: “I think it looks pretty neat. I'd be interested in doing it” *(PF14)*. The negative aspect of the program described by some CWs was that it seemed to resemble other online training programs:

Everything in our training now these days is online. And most people don't even read through it. It's just a matter of going through it and then checking. It's just a checkmark and so I think another online format, would be another issue I think. *(PM6)*

Another participant added: “I think other people see it as another online training that they have to click through…there's no engagement whatsoever. Whereas, if I sat down and talked to somebody in person, it's a much different scenario” *(PM2)*. A suggestion largely expressed by CWs was the inclusion of relevant examples within the therapy modules designed for CWs: “real-life videos of not necessarily actors, but maybe people going through certain situations and talking about certain situations can help,” *(PF4)*. The cartoonish examples in the modules were pointed out by two participants. One participant expressed:

[If cartoonish images are used in modules designed for CWs] They don't feel real. They're kind of cartoony, but then we experience that a lot…it's insulting. Give me a picture of a real person who looks like they're going through distress rather than a cartoonish or screenshotted image of somebody. *(PF4)*

Another participant added:

I do know that most of the training that I did, was all cartoons. There would be some videos that would have definite correctional workers in them and actors, which were funny. But a lot of them, I find that the more serious things tend to be animated. *(PF12)*

When asked if they would prefer real-life examples with CWs integrated into the modules, one CW responded: “I would definitely be more apt to take it if I knew there was information in it from coworkers or other people who have been in corrections,” *(PF12)*.

##### Subtheme: Therapist interaction

Some participants found the text-based platform a limitation: “I find it a lot more difficult and I'm not as inclined to go on because it just feels a lot more like empty typing a note than talking to an actual person” *(PF13)*. Another participant outlined the difference between online and in-person therapy: “Even if it's got great pictures, great stories, great videos to play, I never take the same thing out of it than if someone face-to-face teaches me the information” *(PM2)*. However, the benefits of the virtual therapist were expressed, even if there was an impersonal aspect to it:

I can see how it can feel really impersonal because it's a pre-recorded set of slides but the feedback that you get from your individual therapists, I think is what will make it better for anyone who participates in this. (*PF4)*

One participant suggested adding a virtual face-to-face therapist instead of solely communicating through text:

I can do it [the therapy] on my phone or my laptop or whatever and it gives a little bit more freedom even to go back to the material and read it over again. But again, maybe a weekly session to go along with that, to speak with a therapist and they would kind of review the material with you, might be beneficial as well. Just for somebody that may not have prior knowledge of the terms that you may use or therapy in general, that piece might be beneficial. *(PF16)*

This idea was also expressed by another participant: “I would say maybe having a time where you are on a call, like a zoom call, with your therapist and talking through what you said for that week or two weeks or what not,” *(PF13)*. Another participant included, “I agree with that, or perhaps for the initial assessment and goals, [it] would be helpful to have an in-person call,” *(PF11)*. Another CW suggested having optional therapist contact each week:

I think maybe if there is an option box for each week, where you could say, ‘hey I think I need to talk to someone this week,' or maybe I'm just gonna write my answers, or maybe you can do both. *(PF14)*

Providing homework feedback during virtual meetings instead of textual content was also expressed:

I think for a response, even a 15 min [meeting]. Because you're basically just going to try to convey what you would normally write in that paragraph you sent back. But you're gonna give the person in therapy the option to ask real-time questions and have a quick little conversation, maybe explain things they don't quite understand and just kind of give them the opportunity to have that back and forth. *(PM3)*

These strategies were seen as good ways to develop a good therapeutic relationship:

I really like having an idea of who you're talking to and knowing that you're talking to a person and not just a computer and that you can develop a therapeutic relationship. *(PF4)*

#### Member check

The themes were sent to all study participants (*n* = 21; 10 men and 11 women). Three individuals responded (one man and two women). Non-respondents were assumed to accept that the data were valid and reflected reality. All respondents participated in the gender-specific focus groups and one woman was also present in the individual interviews. These participants agreed with the summarized responses. Another respondent, a man, indicated that the best support they received was from a psychologist who had first-hand experience in a correctional environment.

One of the women also added that not being able to tell the family what happens at work or disclosing too much can be a problem. She included that women experienced emotional and sexual violence. She also mentioned that CWs cannot choose their own therapist through internal programs. Lastly, she suggested being able to download sessions from the platform.

## Discussion

The current study provided insight into the mental health challenges and needs of CWs in Ontario, Canada. Interviews and focus groups explored the lived experiences of CWs, including gender-specific experiences in the workplace and opinions on psychotherapy modules previously designed for the general population and validated by the research team (17–19). The findings suggest the need to reform correctional work culture and reassess the mental health services available to this population.

### Work culture

In line with the current body of research (6), correctional work culture was described as one marked by high stress, trauma, and violence. The findings support role overload as having a large effect on job stress (26). The pervasiveness of job stress was also reported to stem from role boredom and monotony. Hypervigilance, a dominant trait in CWs (27), can be a result of the interaction between monotonous correctional work and chronic trauma exposure (28). The current study provides a novel perspective that inadequate preparation stemming from the multiple roles that CWs take on can worsen sentiment toward workplace monotony. The stress of separating work and personal life identities may also add to the significant work-life conflict observed in CWs (29–31). As a result, detachment and decompressing behaviors were frequently reported by participants. In British prison officers, those who successfully detached themselves from work-related challenges were more likely to have a good work-life balance and experience greater psychological health (30). At the same time, ignoring the work conditions that instill these behaviors can have a pernicious effect on employee mental health and burnout risk in the long term (32, 33).

Although a previous study observed no significant difference in the rate of violence experienced by male and female prison employees (34), the current findings indicate that violence type may be influenced by gender. The experiences of women CWs are in line with concerns of gender inequality and sexism that appear in this male-dominated profession (35). These factors may partly explain why all the women in the study kept their webcams off while all the men kept theirs on. Threats to identity safety may result in women being less vocal in expressing their concerns without anonymity (36, 37). It may have also contributed to the negative views some women CWs expressed toward members of their own gender. At the same time, workplace harassment training programs were not viewed as effective by participants. Similarly, a recent study also demonstrated the ineffectiveness of these training programs (38). Paradoxically, these training programs have been shown to increase the likelihood of victim blaming amongst men due to a defensive reaction (38). In line with the current findings, the study authors suggested manager and leadership training to help detect and de-escalate early signs of harassment. Working with employees to develop actionable tools and strategies may encourage men to have a proactive role in promoting a positive work culture. In turn, this approach could potentially discourage the toxic masculinity expressed by the women CWs in the study.

### Mental health care

As indicated by study participants and supported by other studies (39), organizational and managerial support, mutual trust, and an opportunity to provide constructive feedback on services are effective ways to improve program efficacy and protect against the harmful mental repercussions of correctional work. Although stigma and fears of judgement were expressed by study participants, many highlighted the importance of peer support and being able to talk to someone who understood their experience. Stigma is a key factor in the normalization of toxic masculinity (i.e., socially regressive male traits) and isolation, all of which can contribute to and worsen adverse mental health outcomes and discourage individuals from seeking care (40, 41). Conversely, social support is associated with lower odds of PTSD positive screens in CWs (42). Indeed, study participants described the protective effects of supportive interpersonal relationships with management, colleagues, and family.

Participants also advocated for sector-specific therapy. Previous research on public safety personnel indicates that sector-specific therapists can strengthen therapeutic alliance (43, 44). The findings extend on this by shedding light on the seclusion of this profession and difficulty in explaining work experiences to individuals outside of the profession, including therapists that CWs had worked with. Previous findings also demonstrate that therapists available through institution-provided programs typically lack knowledge of correctional work (45) and their services are less likely to be used for work-related reasons (46). Participants also suggested including real-life examples in therapy that they can better relate to. These examples may be a lesser but beneficial form of social support and an alternative way for CWs to feel connected while refraining from sharing information they perceive will jeopardize their standing in the workplace (47). Many hailed in-person therapy for creating a more personable experience and enabling strong therapeutic connections. Similar to previous findings (48), although participants found the online programs to be convenient, accessible, and time-flexible, many pointed out that the lack of a face-to-face therapist could make the program impersonal. Some suggested including a video component to enhance working alliance.

### Strengths and limitations

The study had several strengths. The combination of individual interviews and focus groups was meant to increase the credibility and validity of the findings (49). The triangulation of individual interviews and the focus group aimed to enhance data richness and provide a more comprehensive understanding of the mental health landscape in corrections (50). In addition, exploring perceptions of digital mental health programs is a unique novelty of the current study. These insights are valuable considering that the use of digital mental health programs is gaining traction in correctional facilities (11, 43, 51). Providing a platform for CW voices can help guide clinical practice surrounding digital therapies for this population. Not only can this strengthen mutual trust and integrity (33, 52), but it can aid in developing programs that acknowledge limitations and barriers expressed by users.

Although the study had many strengths, it also warrants discussing its limitations. The study participants were ethnically homogenous with the majority identifying as white. Since low racial diversity exists in correctional work (53, 54), the current sample may reflect these trends. At the same time, it is important to explore factors that may prevent BIPOC groups from sharing their mental health experiences and needs. Differential outcomes in mental health care preferences and needs of diverse racial and cultural groups (55, 56) suggest consideration of these groups in future studies. Lastly, most of the study participants had a mental health diagnosis and detailed their frustrations navigating through the current care system. Future studies may want to consider the experiences of participants who are not diagnosed or have recovered from their mental diagnoses. These individuals may provide additional perspectives on mental health challenges and services that may not have been previously considered.

## Summary and conclusion

The needs expressed by CWs reflected a culture of mutual trust, where employees feel supported in the workplace and a better relationship with management exists. In general, social connectivity was an important characteristic in CWs and cited as an integral part of therapy and peer support. CWs also expressed the need to work with therapists with expertise in the correctional field and the same belief was extended to digital mental health programs. When presented with samples of previously validated online psychotherapy modules, many participants described their convenience, but suggestions were made to enhance the therapeutic relationship. Taken together, the study demonstrates the importance of considering work culture and mental health needs when developing appropriate programs.

## Data availability statement

The original contributions presented in the study are included in the article/supplementary material, further inquiries can be directed to the corresponding author.

## Ethics statement

The study involving human participants was reviewed and approved by Queen's University Health Sciences and Affiliated Teaching Hospitals Research Ethics Board. The patients/participants provided their verbal informed consent to participate in this study, which was documented by a research assistant on the team.

## Author contributions

EM, NA, and YK were responsible for designing the study and writing the manuscript. The focus groups and interviews were conducted by EM, YZ, AnK, and AlK. EM, YZ, and YK conducted data analysis. Subsequent drafts were edited and finalized by YZ, AnK, AlK, CP, MO, CG, MM, and AFS. All authors contributed to the article and approved the submitted version.

## Funding

This study was funded by the Canadian Institutes of Health Research Operating Grant (File #: RN410776 – 433679). The funding agency has no role in the writing of this paper.

## Conflict of interest

Authors NA and MO cofounded the care delivery platform in use (i.e., OPTT) and have ownership stakes in OPTT Inc.

The remaining authors declare that the research was conducted in the absence of any commercial or financial relationships that could be construed as a potential conflict of interest.

## Publisher's note

All claims expressed in this article are solely those of the authors and do not necessarily represent those of their affiliated organizations, or those of the publisher, the editors and the reviewers. Any product that may be evaluated in this article, or claim that may be made by its manufacturer, is not guaranteed or endorsed by the publisher.
